# A Case of Multinucleate Cell Angiohistiocytoma with New Reflectance Confocal Microscopy Findings

**DOI:** 10.3390/diagnostics12051276

**Published:** 2022-05-20

**Authors:** Mihai Lupu, Tiberiu Tebeica, Ana Maria Malciu, Vlad Mihai Voiculescu

**Affiliations:** 1 Department of Dermatology, MEDAS Medical Center, 030442 Bucharest, Romania; 2 Department of Dermatology, “Carol Davila” University of Medicine and Pharmacy, 050474 Bucharest, Romania; 3 Department of Histopathology, Dr. Leventer Centre, 011216 Bucharest, Romania; tebeica@gmail.com; 4 Department of Dermatology and Allergology, Elias Emergency University Hospital, 011461 Bucharest, Romania; ana.malciu@gmail.com

**Keywords:** confocal microscopy, dermoscopy, multinucleate cell angiohistiocytoma, skin, benign neoplasms

## Abstract

Multinucleate cell angiohistiocytoma (MCAH) is a rare, benign, vascular or fibrohistiocytic tumor usually presenting as single or multiple, reddish-brown papules mostly affecting the limbs and dorsum of the hands of middle-aged females. Since 1985, relatively few MCAH cases have been reported. In vivo reflectance confocal microscopy (RCM) findings of MCAH have never been described. We report a case of MCAH with new non-invasive imaging findings through RCM in correlation with dermoscopy and histopathology. A 66-year-old woman with an unremarkable family and personal history of an atypical nevus presented with a lesion on her right breast. It had appeared 12 months earlier and progressively enlarged. Physical examination revealed a 20 × 11.6 mm, non-tender, reddish-brown maculo-papular lesion with blurred margins. Dermoscopy showed diffusely arranged reddish areas, coalescing whitish patches, truncated and dotted vessels, and a peripheral brown reticulated pattern. RCM revealed a poorly outlined lesion with a normal honeycomb pattern, numerous vessels at the dermal–epidermal junction, and isolated, large, mildly reflective, bizarre structures with angulated edges. These findings correlated well with histological features, which established the diagnosis of MCAH. Even though histopathology remains the gold standard in the diagnosis of MCAH, non-invasive tools such as RCM can help rule out other entities, therefore reducing surgery-associated morbidity.

## 1. Introduction

First described in 1985 by Smith L.P. [1], multinucleate cell angiohistiocytoma (MCAH) is a rare, peculiar, cutaneous entity considered to be a benign, vascular or fibrohistiocytic tumor [2]. Clinically, it presents as single or multiple, red to reddish-brown firm papules initially thought to mostly affect the limbs and dorsum of the hands of middle-aged females. Papules have a tendency to coalesce or take on annular or linear configurations. Sporadic cases have been described on the face or trunk, and one case in the oral mucosa has been reported [3]. The occasional spontaneous regression of MCAH lesions suggests that it may be a reactive process [4].

On dermoscopy, the presence of three types of structures can be typically observed: diffuse reddish areas with blurred edges, more or less scattered whitish patches, and isolated areas of fine peripheral reticulation [5].

Histologically, the lesion is characterized by basket-woven orthokeratosis, a proliferation of dilated capillary dermal vessels, dense collagen in the upper dermis, superficial parallel fibrosis, histiocytes, stellate fibroblasts, and bizarre, angulated multinucleated cells (MCs) [6]. These MCs are not exclusive to MCAH, and may be seen in other lesions such as dermatofibroma (DF) (especially the atrophic vascular variant) and angiofibroma.

In vivo reflectance confocal microscopy (RCM) findings of multinucleate cell angiohistiocytomas have not yet been described. Herein, we report a case of MCAH with dermoscopic and RCM findings, in correlation with histology.

## 2. Case Report

A 66-year-old woman presented to the clinic with a maculo-papular lesion on her right breast. Written informed consent was obtained, and the patient agreed to the proposed diagnostic and therapeutic procedures. The study was conducted in accordance with the Declaration of Helsinki, and the protocol was approved by the Ethics Committee of the “Carol Davila” University of Medicine and Pharmacy Bucharest (Project Number 185/26.12.2018).

**Figure 1:**
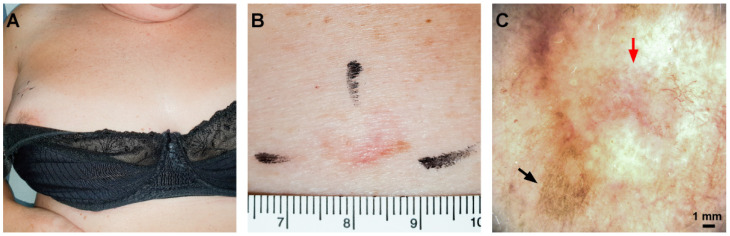
Clinical and dermoscopical aspect of the lesion. (**A**) Solitary, reddish lesion located on the right breast of a 66-year-old woman. (**B**) Close-up image of the lesion in panel A (measuring scale graded in centimeters)—asymmetrical lesion with ill-defined borders and peripheral brown pigmentation. (**C**) Dermoscopically, the lesion was characterized by the presence of a reddish areas, coalescing white patches, clusters of in-focus, short, truncated blood vessels and dotted vessels (red arrow), and a peripheral, brown reticulated pattern (black arrow).

Family history was unremarkable, but the patient had a personal history of a surgically treated atypical nevus and was a long-term smoker. The lesion had appeared one year earlier and slowly enlarged over this period (Figure 1A). Physical examination revealed a 20 × 11.6 mm, non-tender, reddish-brown maculo-papular lesion with clinically blurred margins (Figure 1B) located on the right breast. Dimple sign was negative. Clinical differential diagnosis included dermatofibroma, angiofibroma (fibrous papule) (Table 1), cutaneous lymphoid hyperplasia, granuloma anulare, lichen planus, sarcoidosis, and insect bite.

Dermoscopy showed an asymmetric, ill-defined lesion with diffusely arranged reddish areas, coalescing whitish patches, clusters of in-focus, short, truncated blood vessels, dotted vessels, and a peripheral, fine, brown reticulated pattern (Figure 1C).

**Figure 2:**
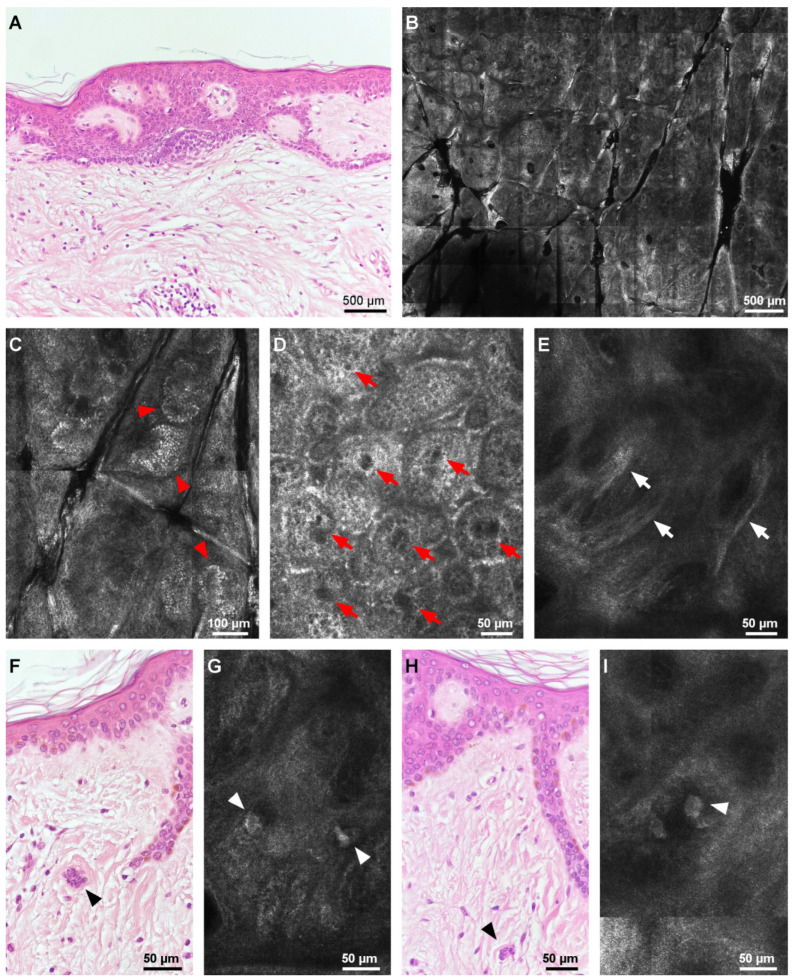
Histopathological and RCM aspects of the lesion. (**A**) Histological architecture of the lesion showing a hyperplastic epidermis, basaloid induction, a proliferation of small vessels, and thickened collagen fibers arranged parallel to the surface, at the level of the reticular dermis (hematoxylin–eosin, 40×). (**B**) RCM mosaic depicting a poorly outlined lesion, presenting with a regular honeycomb pattern and clusters of hyper-reflective cells at the periphery. (**C**) RCM close-up mosaic of the hyper-reflective cells at the periphery (red arrowheads) (correlating histologically to pigmented keratinocytes). (**D**) RCM mosaic showing numerous round, dark spaces at the dermal–epidermal junction (red arrows). (**E**) RCM mosaic at the level of the upper dermis demonstrating hyper-reflective, strand-like structures (white arrows) which correlate to dense collagen bundles parallel to the surface on histology. (**F**,**H**) Histopathology photomicrographs (hematoxylin–eosin) revealing multinucleated cells (black arrowheads) corresponding on RCM (**G**,**I**) to mildly reflective, bizarre structures with angulated edges (white arrowheads).

RCM examination was carried out using a commercially available confocal microscope (VivaScope 1500 Gen 4; MAVIG GmbH, Germany). The microscope uses an 830 nm laser diode to generate in vivo, horizontal, en-face, monochrome images of human skin at quasi-histological resolution. RCM images revealed a poorly outlined lesion, presenting, at the level of the epidermis, a regular honeycomb pattern (Figure 2B) and clusters of hyper-reflective cells at the periphery (Figure 2C). Numerous round, hypo-reflective dark spaces (correlating to blood vessels) were present at the dermal–epidermal junction (Figure 2D). During real-time RCM examination, moderately reflective, small, round structures (correlating to blood cells) could be observed moving through the aforementioned spaces. Hyper-reflective, dense collagen bundles could be seen in the upper dermis (Figure 2E). Scattered throughout the lesion, isolated, large, mildly reflective, bizarre structures with angulated edges were identified at the level of the superficial dermis (Figure 2G,I).

The lesion was surgically excised under local anesthesia. After fixation in formalin, 4 μm thick, vertical sections were prepared from paraffin-embedded blocks and stained with hematoxylin–eosin (H&E). Histological examination on H&E slides revealed a centrally hyperplastic epidermis, basaloid and sebaceous induction, a proliferation of small vessels, and thickened collagen fibers arranged parallel to the surface, at the level of the reticular dermis (Figure 2A). Interstitially, the presence of peculiar, multinucleated cells in the superficial and upper mid-dermis was observed. These cells featured up to 10 hyperchromatic nuclei and a basophilic, angulated, scalloped cytoplasm (Figure 2F,H). The surrounding dermis showed a lymphohistiocytic inflammatory infiltrate. The histological aspect was suggestive of a multinucleate cell angiohistiocytoma.

The patient received no further therapy, and no signs of recurrence were observed 24 months after surgery.

## 3. Discussion

Multinucleate cell angiohistiocytoma is a rare, benign, fibrohistiocytic tumor [2]. Typically, MCAH presents as single or multiple, red to reddish-brown firm papules with a tendency to coalesce or form annular or linear configurations. The most commonly involved sites are the limbs, especially the dorsum of the hands. Less frequent sites include the face and trunk. Middle-aged females are reported as most affected [10]. Aside from the uncommon location on the right breast, our case fits previous descriptions.

Undoubtedly, the predominant dermoscopic finding were reddish, out-of-focus areas due to the lesion’s vascular characteristics, and white patches correlated to thickened dermal collagen. On the other hand, we believe that the isolated peripheral areas with a fine reticulated appearance translate to the presence of melanin in the epidermal ridges, as occurs in dermatofibroma. In this sense, we found certain correlations between some of the dermoscopic findings in this MCAH and those described by Zaballos et al. [11] in an extensive morphological study of dermatofibromas. The observed dermatoscopic structures enable the distinction of MCAH from other vascular or inflammatory entities with which differential diagnosis can be made, and in which other specific patterns have been identified. This is the case of Kaposi’s sarcoma (characteristic rainbow pattern [12]) and lichen planus (Wickham’s striae [13]).

Histopathologically, in MCAH there is a proliferation of dilated dermal vessels, dense collagen in the upper dermis, stellate fibroblasts, and bizarre, angulated multinucleated cells (MCs) [8]. Dermatofibromas more commonly have epidermal hyperplasia, collagen trapping, and more of a storiform growth pattern [8]. Angiofibroma (fibrous papule) also presents with an increased number of dilated capillaries but fewer MCs, and collagen bundles are more vertically oriented and have a perifollicular distribution [8]. Kaposi’s sarcoma does not present with multinucleated cells [8]. Acroangiodermatitis has tortuous, thick-walled blood vessels and a generous hemosiderin deposition, unlike MCAH [8]. Thus, in our case, the constellation of histological features clearly led to the diagnosis of MCAH.

Despite the increasing number of reported cases of multinucleate cell angiohistiocytoma, we have not found any reflectance confocal microscopic description of MCAH while searching the literature. RCM examination showed a normal epidermis with a regular honeycomb pattern and clusters of hyper-reflective cells at the periphery, corresponding to pigmented keratinocytes in the area of dermoscopically visible fine reticulated pattern. At the level of the DEJ and superficial dermis, numerous dilated vessels with obvious blood flow and hyper-reflective rings surrounding dermal papillae, similarly to that in dermatofibroma, could be observed. The upper dermis showed bright, thick, fiber-like structures correlating to white patches on dermoscopy and thickened collagen on histology. Unlike angiofibroma, collagen fibers were not distributed perifollicularly and basal cell carcinoma-like structures were absent. The key histological finding in MCAH was multinucleated angulated cells, which were identified on RCM images as isolated, large, mildly reflective structures with angulated edges at the level of the superficial dermis.

MCAH may spontaneously regress; therefore, treatment is usually not required, but lesions can be successfully removed by surgical excision, cryosurgery, carbon dioxide or argon laser, intense pulsed light, and 585 nm pulsed dye laser [14,15,16].

## 4. Conclusions

Although the confocal features of similar lesions, such as dermatofibroma and angiofibroma, have already been described [9,17], to the best of our knowledge, this is the first reported RCM description of MCAH. We believe that in such cases, non-invasive tools such as dermoscopy and RCM can help to rule out other cutaneous processes prior to lesion biopsy. The relatively small number of reported cases has led to a limited understanding of this entity [10]. Due to its apparent rarity, MCAH may actually be underdiagnosed by pathologists, and should thus remain within the differential diagnosis of practitioners.

## Figures and Tables

**Table 1:** RCM and histological features of multinucleate cell angiohistiocytoma and its mimickers.

Lesion Type	RCM	Histopathology
**Dermatofibroma**	Typical honeycomb pattern, with occasional epidermal streaming. Numerous dilated hypo-reflective spaces (vessels) at the dermal–epidermal junction. Hyper-reflective “rings” composed of monomorphic cells surrounding dark dermal papillae [7].	Circumscribed but unencapsulated tumor predominantly dermal-based. Variable amounts of epidermal hyperplasia, basal layer hyperpigmentation, and follicular induction. Lightly eosinophilic spindle cells present in a focally storiform growth pattern. Secondary elements including Touton-type giant cells, hemosiderin and foam-laden cells, and a lymphohistiocytic inflammatory infiltrate. Collagen trapping at the periphery [8].
**Fibrous papule** **(angiofibroma)**	Regular honeycomb pattern. Nodules with increased cellular density and occasional clefting (BCC-like features). Small, round to linear and canalicular hypo-reflective dark spaces. Hyper-reflective, thickened collagen bundles surrounding hair follicles and BCC-like structures. Small bright round cells [9].	Normal epidermis with epidermal invagination in the papillary dermis or superficial portion of external sheath of the hair follicle. Increased vascularization with variably sized, ectatic blood vessels. Thickened collagen bundles arranged around hair follicles. Occasionally multinucleated cells. Lymphocytes [9].
**Multinucleate cell angiohistiocytoma**	Epidermis with a regular honeycomb pattern and clusters of hyper-reflective cells at the periphery. Numerous round, hypo-reflective spaces (vessels) at the dermal–epidermal junction. Hyper-reflective, dense strand-like structures. Isolated, large, mildly reflective, bizarre structures with angulated edges at the level of the superficial dermis.	Varying epidermal hyperplasia. Proliferation of dilated capillary dermal vessels. Thickened, dense collagen fibers in the upper dermis. Bizarre multinucleated cells with scalloped, angulated cytoplasm. Scattered lymphohistiocytic infiltrate [8].

RCM, reflectance confocal microscopy; BCC, basal cell carcinoma.

## Data Availability

Not applicable.
